# A Ketogenic Diet and the Treatment of Autism Spectrum Disorder

**DOI:** 10.3389/fped.2021.650624

**Published:** 2021-05-11

**Authors:** Qinrui Li, Jingjing Liang, Na Fu, Ying Han, Jiong Qin

**Affiliations:** ^1^Department of Pediatrics, Peking University People's Hospital, Beijing, China; ^2^Department of Pediatrics, Peking University First Hospital, Beijing, China

**Keywords:** autism spectrum disorder, ketogenic diet, neuroprotection, gut microbiota, blood-brain barrier

## Abstract

Autism spectrum disorder (ASD) is characterized by stereotyped behavior and deficits in communication and social interaction. There are no curative treatments for children with ASD. The ketogenic diet (KD) is a high-fat, appropriate-protein, and low-carbohydrate diet that mimics the fasting state of the body and is proven beneficial in drug-resistant epilepsy and some other brain diseases. An increasing number of studies demonstrated that a KD improved autistic behavior, but the underlying mechanisms are not known. We reviewed the neuroprotective role of a KD in ASD, which is likely mediated *via* improvements in energy metabolism, reductions in antioxidative stress levels, control of neurotransmitters, inhibition of the mammalian target of rapamycin (mTOR) signaling pathway, and modulation of the gut microbiota. A KD is likely a safe and effective treatment for ASD.

## Introduction

Autism spectrum disorder (ASD) is a lifelong neurodevelopmental disorder that is characterized by stereotyped behavior and deficits in communication and social interaction. ASD affects 3.4–6.7 per 1,000 children (1). Boys are four times more likely than girls to have ASD (1). The core features of ASD patients are social communication deficits and repetitive sensory–motor behaviors (2). According to the Diagnostic and Statistical Manual of Mental Disorders (DSM) 5, patients who are diagnosed with ASD must have persistent deficits in social communication and repetitive and unusual sensory–motor behaviors (2). Comorbidities in ASD are common and include epilepsy, sleep disorders, gastrointestinal (GI) symptoms, and psychopathologies such as anxiety, depression, attention deficit hyperactivity disorder, and intellectual disability (3). Sleep disturbances occur in 50–80% ASD children, and sleep disorder is associated with behavioral dysregulation (4). Epilepsy is also one of the most common comorbidities in ASD children, and the average prevalence reaches 26% (5). ASD individuals who have epilepsy are likely to exhibit more severe autism-related symptoms (6). GI symptoms, which range from 23 to 70% in ASD children, are related to the severity of ASD (7). Approximately 31% of children with ASD have intelligence quotient scores below 70 (7). The cost of raising a child with ASD is 1.4–3.6 million dollars according to the level of intellectual disability, and the largest expenses are special education costs and the loss of parental productivity. When children grow up, supportive living accommodations and the loss of individual productivity become the highest costs (8). Therefore, ASD places a large burden on society and the affected families. There are no effective drugs for ASD. Several interventions, such as special education and behavioral interventions, provide some benefits, but these interventions do not improve all core symptoms of ASD and have less effects on comorbidities, including epilepsy. Therefore, new therapies are urgently needed to broaden the management options and improve the prognosis of these patients. Lower levels of disaccharidases and hexose transporters were found in ASD patients with GI symptoms (9), which suggests carbohydrate digestion disorders as a physiopathological mechanism in ASD patients. Therefore, a low-carbohydrate diet, such as a ketogenic diet (KD), is likely suitable for ASD patients.

A KD is a high-fat, appropriate-protein, and low-carbohydrate diet that has a positive effect on energy metabolism. For example, a KD increases the levels of adenosine triphosphate (ATP) and enzymes associated with mitochondrial metabolic pathways and enhances mitochondrial biogenesis (10–12). Acetyl-CoA is converted to ketone bodies under the effects of d-β-hydroxybutyrate dehydrogenase, acetoacetate succinyl-CoA transferase, and acetoacetyl-CoA-thiolase (13). Ketone bodies, including β-hydroxybutyrate, acetoacetate, and acetone, function as fuels under fasting or starvation conditions and cross the blood–brain barrier (BBB) to feed the brain. These molecules also prevent mitochondrial permeability transition and attenuate reactive oxygen species (ROS) (14, 15). Therefore, ketone bodies have neuroprotective effects in the central nervous system (CNS). A KD is a significantly effective treatment for epilepsy. Refractory epilepsy patients aged 1–18 years treated with a KD for 4 months had a 56% reduction in mean seizure frequency (16). A KD may also improve some core autistic features and comorbidities of ASD, but data of clinical studies of a KD as a treatment for ASD are very limited. The present review examined the role of a KD in ASD treatment and discussed the underlying mechanisms.

## A Ketogenic Diet and Autism Spectrum Disorder

A KD is a dietary intervention therapy in neurological disorders such as epilepsy and ASD (17, 18). A KD may be an effective therapy for ASD because it might improve ASD core symptoms and could benefit its comorbidities, including seizures. The efficiency of a KD must be monitored using urinary ketones and serum beta-hydroxybutyrate (BHB) (19, 20). Some evidence showed that a KD improved the core features of ASD patients (Table 1). El-Rashidy et al. showed that a KD improved autistic manifestations, which was demonstrated as improved scores on the Autism Treatment Evaluation Test (ATEC) scales and the Childhood Autism Rating Scale (CARS), especially sociability improvement (19). Lee et al. also reported that a modified ketogenic gluten-free diet with supplemental medium-chain triglycerides (MCTs) improved the social affect subdomain and total autism diagnostic observation schedule, 2nd edition (ADOS-2) scores, but it did not affect the restricted and repetitive behavior scores (20). A KD improved social exploration and social interactions in an animal model of ASD (26, 27). It also ameliorates the comorbidities of ASD more efficiently than the core symptoms of ASD. A KD improved the social communication of one of six ASD patients, but it reduced the comorbidities of all six ASD patients, including attention deficit hyperactivity disorder (ADHD), compulsive behavior, preoccupation with parts of objects, and abnormal sleep (23). It also decreased the frequency of seizures (28). A case report of an ASD child found that a KD improved the electroencephalogram results and increased the child's intelligence quotient (21). Although a KD-induced decrease in seizures will lead to a better quality of life in patients with epilepsy, including ASD, it is not associated with improvements in behaviors in ASD patients. Antiepileptic drugs do not have a large effect on the behavioral symptoms in ASD (29).

**Table 1:** Treatment with a modified KD and outcomes in ASD patients.

**Object**	**Diet**	**Duration**	**Outcome**	**Reference**
A child with autism and epilepsy	Gluten-free, casein-free diet, then KD added	14 months	Improved cognitive and social skills, language function, and stereotypies and reached seizure-free status	(21)
A 6-year-old child	Ketogenic diet	16 months	Improved behavior and intellect; ^18^F-FDG uptake decreased in whole cortex.	(22)
15 children aged 2–17 years	A modified ketogenic gluten-free diet with medium-chain fatty acids	3 months	Improved core autism features. No difference in restricted and repetitive behavior scores	(20)
45 children aged 3–8 years	Group 1: ketogenic dietand modified Atkins diet (MAD) Group 2: gluten-free casein-free (GFCF) diet Group 3: control group	6 months	Groups 1 and 2 showed improvement in ATEC and CARS scores.	(19)
Six ASD patients with a pathological increase in beta-hydroxybutyrate	Ketogenic diet	–	One patient showed a remarkable improvement in the CARS scale (41 → 21). Another patient had subtle clinical improvements.	(23)
30 children aged 4–10 years	Ketogenic diet	6 months	18/30 had an improvement in CARS.	(24)
A developmentally severely disabled man	Modified Atkins diet	1 year	Epilepsy settled, autistic features were alleviated, and behavioral problems disappeared	(25)

*ASD, autism spectrum disorder; KD, ketogenic diet; CARS, Childhood autism rating scale; ATEC, autism treatment evaluation test*.

After KD treatment, some blood parameters changed, such as an upregulation of beta-hydroxybutyrate, high-density lipoprotein (HDL), low-density lipoprotein, and cholesterol and a downregulation of eosinophils (20). Only the HDL increase, eosinophil percentage, and white blood cell count decrease predicted the effects of a modified KD treatment in ASD patients (20). Why the effects of KD vary so much from patient to patient in ASD is not clear. The pro-inflammatory condition at baseline was associated with an effective KD treatment, which was demonstrated by a reduction in eosinophils (20).

A KD seems effective in ASD patients, but all of these clinical studies had small sample sizes, which is likely due to the difficulty of setting up randomized trials in ASD children. ASD patients also eat a narrower range of foods and exhibit more feeding problems. They refuse some food because of the presentation or the need for certain utensils (30). Therefore, it is difficult to introduce the KD to ASD children. The duration of these studies was 3–16 months, which is not sufficient to assess the side effects of KD. In summary, more studies are needed to verify the precise role of KD in ASD patients.

## The Neuroprotective Role of a KD in the Central Nervous System

**Figure 1:**
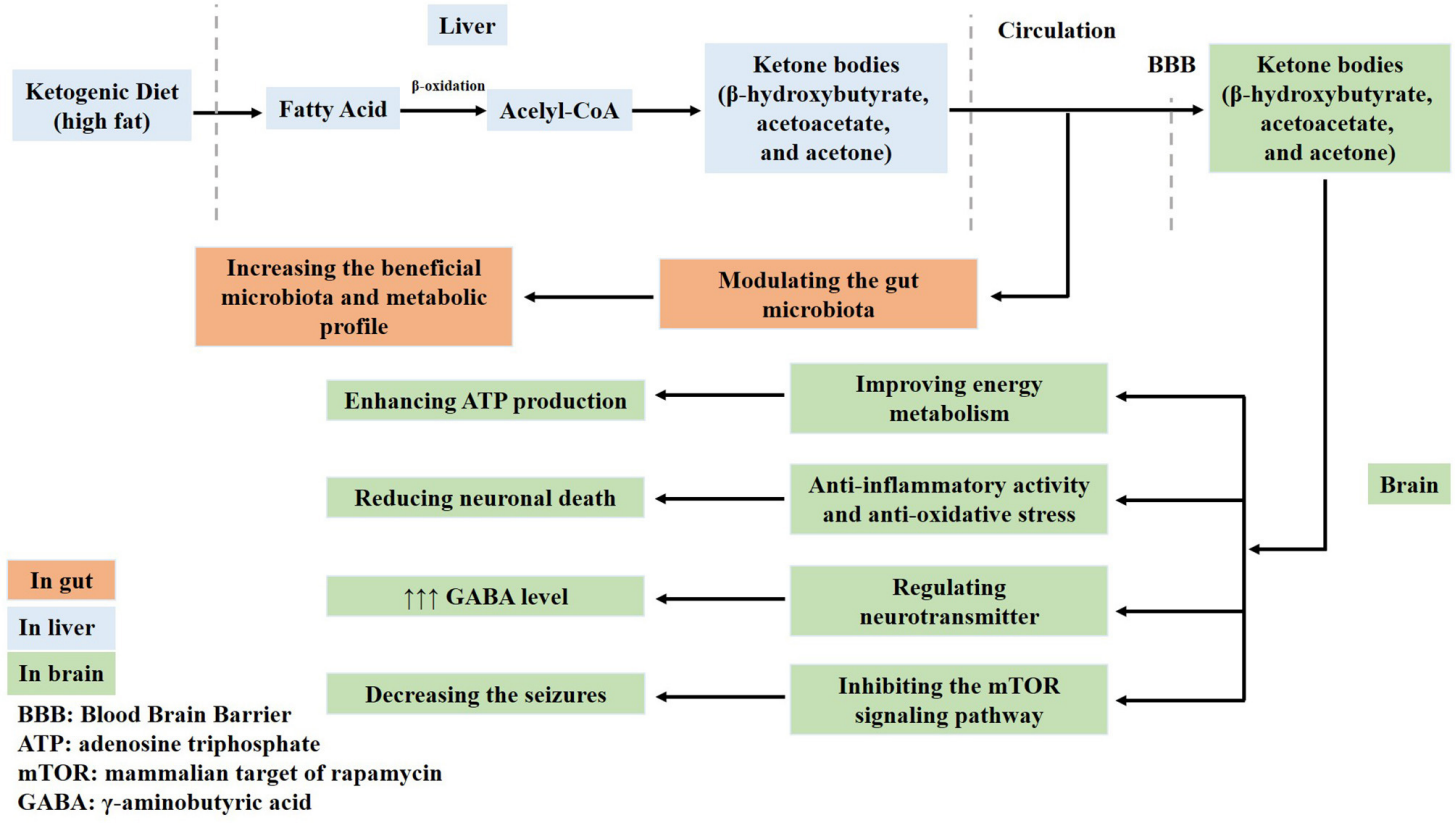
Ketone bodies have neuroprotective effects in the brain. Ketone bodies containing acetoacetate, β-hydroxybutyrate, and acetone generated from a ketogenic diet cross the blood–brain barrier. Firstly, ketone bodies improve the energy metabolism and enhance adenosine triphosphate (ATP) production. Secondly, they normalize mitochondrial function by stimulating mitochondrial biogenesis and reducing oxidative stress, which reduces neuronal death. Thirdly, ketone bodies regulate neurotransmitters and increase γ-aminobutyric acid (GABA) levels. Fourthly, they also inhibit the activation of the mammalian target of rapamycin (mTOR) signaling pathway and decrease seizures. Lastly, ketone bodies modulate the gut microbiota.

A KD contains abundant fat and induces the generation of acetyl-CoA in the mitochondria of the liver *via* fatty acid oxidation. Therefore, abundant acetyl-CoA is shunted to the formation of ketone bodies (acetoacetate, β-hydroxybutyrate, and acetone) in the liver. These ketone bodies enter into the circulation and are used to produce energy. One of the main ketones is acetone, which increases the seizure threshold and potentiates the anticonvulsant activity of some antiepileptic drugs (31). Therefore, fatty acids and ketone bodies exert neuroprotective effects in the brain. For example, ketone bodies improve the energy metabolism by enhancing ATP production and normalizing mitochondrial function *via* the stimulation of mitochondrial biogenesis and the reduction of oxidative stress, which reduces neuronal death. Ketone bodies regulate neurotransmitters, increase γ-aminobutyric acid (GABA) levels, and inhibit the activation of the mammalian target of rapamycin (mTOR) signaling pathway (32–36) (Figure 1). However, the exact neuroprotective mechanisms of a KD are not fully understood.

### The Improvement of Energy Metabolism in the CNS

ASD individuals have impaired mitochondrial energy production due to the presence of abnormal mitochondrial markers in their plasma, such as elevated levels of lactic acid and pyruvate (37). Weissman et al. showed that ASD patients had mitochondrial electron transport chain dysfunction, including complex I and complex III deficiencies (38). A KD provides fuel sources in the human body, and the ketone bodies, including β-hydroxybutyrate, cross the BBB and replace glucose as fuel for the brain. This molecule crosses the BBB *via* proton symporters and a sodium-dependent monocarboxylate transporter, which is located in the BBB, neurons, and astrocytes (39). Ketone bodies enhance adenosine triphosphate (ATP) production *via* the Krebs cycle to generate energy and balance metabolism (39). A KD reduced seizures by enhancing brain metabolism *via* the regulation of transcripts encoding energy metabolism enzymes or mitochondrial proteins in rats with seizures (10).

### Anti-inflammatory Activity and Antioxidative Stress

One of the risk factors for ASD in children is abnormal maternal immune activation. For example, pregnant mice were injected with double-stranded RNA (dsRNA) poly (I: C) to mimic a viral infection, and the offspring of these mice had ASD-like behaviors (40). ASD patients also experience aberrant inflammation. Some cytokines and chemokines, such as interleukin 6 (IL-6), tumor necrosis factor alpha (TNF-α), and monocyte chemotactic protein-1 (MCP-1), are found at abnormal levels in brain samples and cerebrospinal fluid (CSF) in ASD patients (41). These cytokines activate the immune response *via* the nuclear factor kappa-light-chain-enhancer of activated B cells (NF-κB) signaling pathway (42). Young and colleagues found that the NF-κB protein was hyper-expressed in mature microglia in brain samples in ASD patients, which indicates that immunity is activated in the brains of ASD patients (43). ASD patients also had higher levels of eight cytokines in plasma compared to control individuals (44). ASD individuals also have associated oxidative stress level upregulation and antioxidant ability downregulation. Evidence showed that antioxidant enzymes, including superoxide dismutase and glutathione peroxidase, are altered in ASD patients, which increases inflammation (45). In summary, there is a consensus that ASD individuals have immune dysfunction.

A KD has powerful anti-inflammatory activity and antioxidative stress effects in the brain. Jeong et al. found that a KD reduced neuroinflammation *via* the activation of the peroxisome proliferator-activated receptor gamma (PPARγ) and protected against excitotoxicity-induced neuronal cell death (46). Fatty acids activate PPARs and are critical regulators of lipid metabolism (47). Greco et al. found that ketone bodies decreased oxidative stress and improved the mitochondrial respiratory complex activity in a traumatic brain injury animal model (48). A KD likely normalizes mitochondrial function by stimulating mitochondrial biogenesis, decreasing oxidative stress and the levels of pro-apoptotic factors, preventing changes in mitochondrial permeability, and decreasing the mitochondrial ROS production in neocortical neurons (14, 49, 50). Mirza et al. showed that rats treated with propionic acid exhibited social impairment and repetitive behavior. The cerebellum, brainstem, and prefrontal cortex of these rats had high levels of oxidative stress and inflammation, with increased IL-6 and TNF-α levels and decreased IL-10 levels. They also found that decreased levels of oxidative stress and inflammation improved neurobehavioral disorders in rats (51).

### The Regulation of Neurotransmitters in the Brain

GABA is the main inhibitory neurotransmitter in the brain, and it originates from glutamate decarboxylation. Glutamate is the primary excitatory neurotransmitter in the CNS. Patients with autism exhibited abnormal levels of proteins and messenger RNAs (mRNAs) associated with the glutamate system in the cerebellum (52). The medium-chain fatty acids that are present during the consumption of a KD directly inhibit glutamate receptors and reduce seizures (53). Other studies found that beta-hydroxybutyrate, which is produced from a KD, inhibited GABA degradation in astrocytes (54). One study showed that children with ASD had reduced GABA levels in sensorimotor function, and this phenomenon was associated with poor tactile performance compared to healthy children (55). ASD patients had significantly lower GABA concentrations in the auditory cortex. Patients with ASD also had abnormal maturation of the neuronal circuitry on magnetoencephalography (MEG) and edited magnetic resonance spectroscopy (MRS) (56). In summary, a KD may ameliorate ASD behaviors *via* the modulation of neurotransmitters, such as increasing GABA levels.

### Inhibition of the mTOR Signaling Pathway

The mTOR signaling pathway is associated with protein synthesis, cell growth, cell proliferation, and axonal sprouting (57). This pathway plays a role in ASD (58–60). Ketone bodies may inhibit the mTOR pathway and exert an anticonvulsant effect (36). Tang et al. found that the mTOR signaling pathway was overactive both in postmortem ASD samples and TSC2^+/−^ mice, and the mTOR inhibitor rapamycin corrected ASD-like behaviors (59). However, eIF4E, which is downstream of mTOR, was associated with ASD-like phenotypes in mice (61). A KD played an anticonvulsant role by decreasing downstream mTOR signaling in the hippocampus of rats with kainate-induced epilepsy (36).

### Modulation of the Gut Microbiota

GI symptoms, including constipation and diarrhea, are common in ASD individuals and are associated with the severity of ASD symptoms (7, 62). Gorrindo et al. reported that constipation was associated with increased social impairment and language disorders (62). Patients with ASD have different gut microbiome components and metabolic products (7). The gut microbiota communicates with the brain *via* the neuroendocrine, neuroimmune, and autonomic nervous systems, which is the so-called microbiota–gut–brain axis (7, 63). Porphyromonadaceae, Prevotellaceae, Bacteroidales, and Lachnospiraceae were more abundant in the ASD animal model (40). An increasing number of studies showed that gut microbiota disturbances were associated with ASD patients, and modulation of the gut microbiota improved symptoms in ASD patients (7, 64, 65).

A KD restored gut microbial composition (66) and improved ASD core features, including social communication and repetitive behaviors, in an ASD animal model (26, 67). A KD increased the levels of *Akkermansia, Parabacteroides, Bacteroides*, and *Desulfovibrio* spp. in animal models of seizure, glucose transporter 1 deficiency syndrome, and ASD (66, 68–70). A KD did not change locomotor activity, anxiety-related behaviors, recognition memory, or sociability in young male rats, which suggests that a KD may be more effective in females in some cases (71, 72). As mentioned above, a KD likely modifies the composition of the gut microbiota in different animal disease models, including ASD. However, there are few studies on alterations of the gut microbiota in humans treated with a KD. In summary, modulation of the gut microbiota may be a new target for therapy in ASD patients.

## The Side Effects of a Ketogenic Diet

ASD children have feeding problems because most of them are selective eaters (30). Therefore, it is difficult to apply a KD to children with ASD. There are also some side effects of the long-term implementation of a KD in children. The main side effects of KD treatment in children are constipation, vomiting, lack of energy, and hunger (73). Late-onset adverse effects include hyperuricemia, hyperlipidemia, and kidney stones (16). One serious side effect of a KD in children is the suppression of physical development (74). Long-term KD administration decreased growth, as indicated by height *z*-scores, but growth, as indicated by weight *z*-scores, did not change. A KD may cause height deceleration (74). However, these side effects do not occur often, and a KD has been widely used in children with refractory epilepsy. In summary, although studies with larger samples of ASD patients are lacking, KD is a safe and effective treatment in people with ASD.

## Conclusions

ASD is a neurodevelopmental disease, and timely and effective treatment help improve the prognosis. There is no effective treatment for ASD children. Many treatments are used for ASD, but there are no curative treatments for all of the core features of ASD. A KD may improve social behavior in ASD *via* normalizing GABA, improving mitochondrial function, ameliorating inflammatory activity and oxidative stress in the brain, inhibiting the mTOR signaling pathway, and modulating the gut microbiota. However, the effects of KD vary widely between ASD patients, and the underlying mechanisms are not known. ASD children may also reject KD food because of their selective eating habits, which complicates the introduction of a KD to ASD patients. Some studies showed that ASD children have nutrient deficiencies, including vitamin D and folic acid (75). Long-term treatment of a KD likely aggravates the nutritional deficiency. The benefits of a long-term KD treatment in ASD children are not known. Therefore, more studies with larger samples and long-term KD treatment are needed to demonstrate the beneficial effects of a KD and its side effects in children with ASD.

## Author Contributions

QL wrote the manuscript. JL and NF reviewed the literature and contributed to writing the manuscript. YH and JQ conceived the review and provided final approval of the version to be published. All authors contributed to the article and approved the submitted version.

## Conflict of Interest

The authors declare that the research was conducted in the absence of any commercial or financial relationships that could be construed as a potential conflict of interest.
